# Motor function and perception in children with neuropsychiatric and conduct problems: results from a population based twin study

**DOI:** 10.1186/1866-1955-6-11

**Published:** 2014-05-20

**Authors:** Peik Gustafsson, Nóra Kerekes, Henrik Anckarsäter, Paul Lichtenstein, Christopher Gillberg, Maria Råstam

**Affiliations:** 1 Child and Adolescent Psychiatry, Department of Clinical Sciences, Lund University, Baravägen 1C, SE-222 40 Lund, Sweden; 2 CELAM (Centre for Ethics, Law and Mental Health), University of Gothenburg, Gothenburg, Sweden; 3 Swedish Prison and Probation Services, R & D Gothenburg, Gothenburg, Sweden; 4 Department of Medical Epidemiology and Biostatistics, Karolinska Institutet, Stockholm, Sweden; 5 Gillberg Neuropsychiatry Centre, Sahlgrenska Academy, University of Gothenburg, Gothenburg, Sweden

**Keywords:** Conduct Disorder, Attention-Deficit/Hyperactivity Disorder, Autism Spectrum Disorder, Motor control, Perception, A-TAC

## Abstract

**Background:**

Children with early symptomatic psychiatric disorders such as Attention-Deficit/Hyperactivity Disorder (ADHD) and Autism Spectrum Disorder (ASD) have been found to have high rates of motor and/or perception difficulties. However, there have been few large-scale studies reporting on the association between Conduct Disorder (CD) and motor/perception functions. The aim of the present study was to investigate how motor function and perception relate to measures of ADHD, ASD, and CD.

**Methods:**

Parents of 16,994 Swedish twins (ages nine and twelve years) were interviewed using the Autism-Tics, ADHD and other Comorbidities inventory (A-TAC), which has been validated as a screening instrument for early onset child psychiatric disorders and symptoms. Associations between *categorical* variables of scoring above previously validated cut-off values for diagnosing ADHD, ASD, and CD on the one hand and motor and/or perception problems on the other hand were analysed using cross-tabulations, and the Fisher exact test. Associations between the *continuous* scores for ADHD, ASD, CD, and the subdomains Concentration/Attention, Impulsiveness/Activity, Flexibility, Social Interaction and Language, and the categorical factors age and gender, on the one hand, and the dependent dichotomic variables Motor control and Perception problems, on the other hand, were analysed using binary logistic regression in general estimated equation models.

**Results:**

Male gender was associated with increased risk of Motor control and/or Perception problems. Children scoring above the cut-off for ADHD, ASD, and/or CD, but not those who were ‘CD positive’ but ‘ADHD/ASD negative’, had more Motor control and/or Perception problems, compared with children who were screen-negative for all three diagnoses. In the multivariable model, CD and Impulsiveness/Activity had no positive associations with Motor control and/or Perception problems.

**Conclusions:**

CD symptoms or problems with Impulsiveness/Activity were associated with Motor control or Perception problems only in the presence of ASD symptoms and/or symptoms of inattention. Our results indicate that children with CD but without ASD or inattention do not show a deviant development of motor and perceptual functions. Therefore, all children with CD should be examined concerning motor control and perception. If problems are present, a suspicion of ADHD and/or ASD should be raised.

## Background

Children with Autism Spectrum Disorders (ASD) and Attention-Deficit/Hyperactivity Disorder (ADHD) have been shown to have high rates of motor and perception difficulties in controlled studies [1-4]. Before the introduction of the concept of ADHD, other diagnostic terms, such as Minimal Brain Dysfunction/Damage (MBD), were used to cover children with attention deficit, hyperactivity, and impulsivity. During the 1950s, MBD was believed to result from multiple small brain lesions and was often considered to be reflected as motor and perception difficulties [5,6]. MBD [6] was often regarded as a form of ‘minimal cerebral palsy’. The currently used diagnosis of ADHD is defined in the *Diagnostic and Statistical Manual of Mental Disorders, 4th edition* (DSM-IV) [7]. DSM-IV lists nine criteria for inattention and nine criteria for hyperactivity-impulsivity (Table 1). To be diagnosed with ADHD, six or more of the criteria for inattention and/or six or more of the criteria for hyperactivity-impulsivity should be fulfilled.

**Table 1:** **Criteria for Attention-Deficit/Hyperactivity Disorder (ADHD) according to** ***Diagnostic and Statistical Manual of Mental Disorders, 4th edition*** **(DSM-IV)**

**Inattention:**	
•	Often fails to give close attention to details or makes careless mistakes in schoolwork, work or other activities
•	Often has difficulty sustaining attention in tasks or play activities
•	Often does not seem to listen when spoken to directly
•	Often does not follow through on instructions and fails to finish schoolwork, chores or duties in the workplace
•	Often has difficulty organizing tasks and activities
•	Often avoids, dislikes, or is reluctant to engage in tasks that require sustained mental effort
•	Often loses things necessary for tasks and activities
•	Is Often easily distracted by extraneous stimuli
•	Is Often forgetful in daily activities
**Hyperactivity/Impulsivity:**	
•	Often fidgets with hands or feet or squirms in seat
•	Often leaves seat in classroom or in other situations in which remaining seated is expected
•	Often runs about or climbs excessively in situations in which it is inappropriate
•	Often has difficulty playing or engaging in leisure activities quietly
•	Is Often ‘on the go’ or Often acts as if ‘driven by a motor’
•	Often talks excessively
•	Often blurts out answers before questions have been completed
•	Often has difficulty awaiting turn
•	Often interrupts or intrudes on others

In the 1980s, Gillberg and Gillberg [8] introduced the concept of Deficits in Attention, Motor control and Perception (DAMP) to describe the coexistence/‘comorbidity’ of ADHD and motor and/or perception problems, later often subsumed under the label of Developmental Coordination Disorder (DCD) [7]. The diagnostic label of DCD is applied to individuals who are impaired by motor coordination difficulties that interfere significantly with their academic achievements or activities in everyday life. Several Swedish studies have shown that about half of all children with ADHD also meet the criteria for DCD and that these children have poorer outcomes than those with either ADHD only or DCD only [9-11]. Children with ADHD comorbid with DCD have, for instance, more cognitive (including language) problems and autistic symptoms, than children with ADHD without DCD [12]. Children with ASD have also been shown to have an increased risk of motor dysfunction/DCD and perception dysfunction [13-19]. Moruzzi *et al*. [20] have shown additive genetic influences on clumsiness and autistic traits in a twin study. Sensory-motor problems, specifically perception-action coupling and temporal control in early infant development, have recently been suggested to be a key component in the progression of ASD [21]. In the *Diagnostic and Statistical Manual of Mental Disorders, 5th edition* (DSM-5) [22], sensory items have been added to the criteria for ASD. In a recent review article, Rogers *et al*. [23] describe a large number of human and animal studies providing support for the hypothesis that individuals with ASD might have cerebellar dysfunction and that the variability in the degree of such dysfunction is likely to be related to the severity of motor deficits in ASD.

Conduct Disorder (CD) is very common among children with ADHD [24] and not uncommon among children with ASD [25]. The most important predictors of CD are environmental factors such as maladaptive punitive parenting and parental antisocial behaviour [26-28]. Genetic factors have also been shown to be of importance for CD [29]. CD and ADHD have been shown to be associated [30]. Martin *et al*. [31] have shown a positive association between hyperactivity and CD. Children with ADHD who develop CD at an early age (mostly boys) are a clinically important group who have a high risk of developing persistent antisocial behaviour during adolescence [8,24].

Given the relationship between ADHD and CD, and the motor and/or perceptual dysfunction in ADHD, it seems likely that some of these symptoms would be present in CD. However, some questions remain as to whether only those children with co-occurring ADHD and CD would demonstrate these motor and/or perceptual problems, as well as the clinical significance of these symptoms in CD. We have only found a few studies describing the association between CD on one hand and motor and/or perceptual dysfunction on the other. In a population based follow-up study, Moffitt and Silva assessed children of 13 years of age and compared those with and without self-reported delinquency and with and without attention deficit disorder [32]. Children with self-reported delinquency had more visuospatial problems and problems with visuo-motor integration than control children. Among children with self-reported delinquency, the children with attention deficit disorder had more problems with visuo-motor integration than children without attention deficit disorder. Raine *et al*. [33] studied young adults with early neuromotor measures collected during the first year of life. They found that subjects with a combination of early neuromotor deficits and unstable family environment had the highest risk of showing violent and criminal behaviour, but they did not compare individuals with or without attention deficit disorder. In a later study [34], Raine *et al*. showed that individuals with persistent antisocial behaviour had a higher frequency of problems with spatial ability assessed at three-years of age compared to controls and individuals with non-persistent antisocial behaviour, and they made a hypothesis that early visuospatial (right hemisphere) impairments could interfere with early attachment, emotion recognition and emotional regulation leading to antisocial behaviour. In a study of criminal offenders, Raine *et al*. [35] showed that spatial as well as verbal impairments among these individuals were not completely attributable to ADHD. In a study concerning difficulties with executive planning of motor output and verbal ability among boys aged six to twelve years, Nigg *et al*. [36] found that these difficulties were better explained by ADHD-problems than by CD or reading disability, although they did not examine a pure group with CD without ADHD.

Clearly, more research is needed concerning the association between CD and motor problems/DCD and perception difficulties. Large-scale general population studies provide an excellent vehicle for studying such possible associations. In an earlier published article, we reported results including the heritability of motor problems, perception, ADHD, ASD, and CD from a large Swedish twin study [37].

The objectives of the present study were to examine the distribution of problems regarding motor function and perception and their relation to childhood neuropsychiatric and behavioural problems in a large population-based twin study, taking gender and age into consideration. We wanted to test the hypothesis that there is an association between CD and motor and/or perceptual problems even among children with CD without ADHD or ASD. We also wanted to test the hypothesis that ADHD (with its subtypes attention deficit and hyperactivity/impulsivity) and ASD (with its three components: rigid fixated behaviour, difficulties with social interaction and difficulties with communication) all are associated with motor and/or perceptual problems.

## Methods

### Subjects

The Child and Adolescent Twin Study in Sweden (CATSS) is an on-going longitudinal twin study targeting all twins born in Sweden since 1 July 1992. Parents of twins are interviewed regarding their children’s somatic and mental health and social environment in connection with the children’s ninth or twelfth birthdays (CATSS-9/12). By January 2010, 8,610 parental interviews concerning 17,220 twins had been completed, with an overall response rate of 80% of all contacted families [37]. If one or both twins in a pair were affected with a severe brain disorder (documented brain damage, prevalence 0.9%, most commonly cerebral palsy) or known chromosomal aberration (prevalence 0.1%, most commonly Down’s syndrome), then that pair was excluded, leaving 16,994 children (8,666 boys and 6,439 girls). Of these, 10,555 were 9-year-olds, and 6,439 were 12-year-olds. Many of the twins were born prematurely; 29% had received neonatal care due to premature birth. Of the children included in the study, 0.5% had a diagnosis of mental retardation and further 0.5% attended a special school for developmentally delayed children.

### The Autism-Tics, ADHD and other Comorbidities inventory

The Autism-Tics, ADHD and other Comorbidities (A-TAC) inventory used in the interviews was developed (originally for use in the Swedish twin study) with a view to screening for all types of child and adolescent psychiatric disorders. The items in the A-TAC are organised in theoretically defined modules based on DSM-IV categories and criteria. For each question the answer ‘no’ is coded as ‘0’ , ‘yes, to some extent’ is coded as ‘0.5’ and ‘yes’ is coded as ‘1’. The A-TAC has been validated in several studies and has been shown to be a sensitive screening tool for ASD, ADHD, tics, learning disorders, and DCD [38,39]. It has been shown to function equally well in both genders (Kerekes N, Lundstrom S, Chang Z, Tajnia A, Jern P, Lichtenstein P, Nilsson T, Anckarsäter H; Oppositional defiant disorder and conduct disorder: neurodevelopmental predictors and genetic background in boys and girls in a nationwide twin study, submitted).

The ‘Motor control problem’ scale of the A-TAC initially comprised five items. In one of the validation studies, the item: ‘does he/she have problems with smooth coordination of movement?’ was found to have the same level of predictive power for the clinical diagnosis of DCD as did all the five items taken as a module score [38]. This single item was therefore used in the present study to screen for ‘Motor control problems’. A cut-off of ≥ 0.5 was used to define ‘DCD caseness’ (yielding a sensitivity of 0.59 and specificity of 0.85 in the validation study) [38].

The Perception scale of the A-TAC comprises five items, organised into one module. These five items are: ‘Does he/she seem disturbed by height differences such as in connection with climbing stairs, and so on?’ , ‘Does he/she have difficulty judging distance or size?’ , ‘Is he/she oversensitive to the touch of tight clothing?’ , ‘Is he/she particularly sensitive to certain sounds/noise?’ , and ‘Is he/she particularly sensitive to certain flavours, smells, or consistencies?’ Thus, the item ‘perception’ in the A-TAC contains questions concerning visual, auditory, sensory, olfactory and gustatory perception and, to some degree, proprio-sensibility (climbing stairs). To define ‘Perception disorder caseness’ , a cut-off of ≥ 2.5 points was used (yielding a sensitivity of 0.62 and a specificity of 0.93 in the validation study [38]). The internal consistency for the perception scale measured by Cronbach’s α has been shown to be 0.62.

The ADHD scale of the A-TAC consists of 19 items organised into two modules/subdomains: Concentration/Attention and Impulsiveness/Activity. The scale has strong internal consistency [37] and excellent predictive ability [38]. ‘ADHD caseness’ was defined by the validated cut-off ≥ 6.0 points on the ADHD scale (yielding a sensitivity of 0.98 and a specificity of 0.81 in the validation study). The internal consistency for the ADHD scale (Cronbach’s α) is 0.92.

The ASD scale of the A-TAC consists of 17 items organised into three modules/subdomains: Language, Social Interaction and Flexibility. The scale has both excellent internal consistency and predictive ability [37,38]. ‘ASD caseness’ was defined in accordance with the validated cut-off ≥ 4.5 points on the ASD scale (yielding a sensitivity of 0.96 and a specificity of 0.88 [38]). The internal consistency (Cronbach’s α) for the ASD scale is 0.86.

The CD scale of the A-TAC consists of five items, with good internal consistency [37] and excellent predictive ability. ‘CD caseness’ was defined here as a score of ≥ 2.0 (with a sensitivity of 0.55 and a specificity of 0.98) (Kerekes N, Lundstrom S, Chang Z, Tajnia A, Jern P, Lichtenstein P, Nilsson T, Anckarsäter H; Oppositional defiant disorder and conduct disorder: neurodevelopmental predictors and genetic background in boys and girls in a nationwide twin study, submitted). The internal consistency (Cronbach’s α) of the CD scale is 0.61.

The main modules/subdomains of the A-TAC scales were also analysed as continuous scale variables.

### Statistical analysis

The associations of ADHD, ASD, or CD caseness on the one hand, and DCD and Perception disorder caseness on the other hand, were analysed using the Fisher exact test (two-way, three-way and four-way cross tabulations). The associations of defined continuous covariates and categorical cofactors with DCD and Perception disorder were studied using binary logistic regression analyses in General Estimated Equation (GEE) models, and the relationship between the twins in each pair was taken into consideration. DCD and Perception disorder constituted the dichotomous dependent variables. Gender as a co-factor had significant odds ratios (ORs) for DCD and Perception disorder; hence GEE models were analysed by gender. The Fisher r-to-z transformation was used to test the significance of the difference between the Spearman correlations for prematurely born and full term children.

### Ethical consideration

The CATSS-9/12 study has ethical approval from the Karolinska Institute Ethical Review Board: Dnr 03–672 and 2010/507-31/1.

## Results

### Prevalence of DCD and Perception disorder caseness by ADHD, ASD and CD status

ADHD, ASD and, CD caseness was associated with much higher rates of DCD and Perception disorder caseness (Table 2). CD caseness in itself (without concomitant ADHD or ASD caseness) was not associated with DCD or Perception problems (Table 3). DCD caseness and Perception disorder caseness were much more common in ADHD plus ASD caseness (55% and 24%) than in ADHD caseness ‘only’ (18% and 3%, *P* < 0.001). Perception disorder caseness was also somewhat more common in ADHD caseness plus CD caseness than in ADHD caseness ‘only’ (5% versus 3%, *P* < 0.05).

**Table 2:** Cross tables showing the frequencies of Motor problems and Perception problems among children screen-positive for Attention-Deficit/Hyperactivity Disorder (ADHD), Autism Spectrum Disorder (ASD) and Conduct Disorder (CD)

	**ADHD screen-negative cases**	**ADHD screen-positive cases**	**ASD screen-negative cases**	**ASD screen-positive cases**	**CD screen-negative cases**	**CD screen-positive cases**
No Motor problems	14,449	1,218	15,426	249	15,143	530
	94.4%	73.1%	93.7%	47.6%	92.80%	79.70%
Motor problems	854	448	1,034	274	1,177	135
	5.6%	26.9%	6.3%	52.4%	7.2%	20.3%
Total	15,303	1,666	16,460	523	16,320	665
	100%	100%	100%	100%	100%	100%
Cross-table significance	*P* < 0.001		*P* < 0.001		*P* < 0.001	
	**ADHD screen-negative cases**	**ADHD screen-positive cases**	**ASD screen-negative cases**	**ASD screen-positive cases**	**CD screen-negative cases**	**CD screen-positive cases**
No Perception problems	15,230	1,478	16,369	350	16,135	585
	99.5%	88.7%	99.4%	66.9%	98.9%	88.0%
Perception problems	73	188	91	173	185	80
	0.5%	11.3%	0.6%	33.1%	1.1%	12.0%
Total	15,303	1,666	16,460	523	16,320	665
	100%	100%	100%	100%	100%	100%
Cross-table significance	*P* < 0.001		*P* < 0.001		*P* < 0.001	

Column percentages are shown. Significances have been calculated according to Fisher’s exact test.

**Table 3:** Cross tables showing the frequencies of Motor and Perception problems among children in the following four categories: 1. Cases screen-negative for Conduct Disorder (CD); 2. Cases screen-positive for CD; 3. Cases screen-negative for Attention-Deficit/Hyperactivity Disorder (ADHD), Autism Spectrum Disorder (ASD) and CD, and 4. Cases screen-negative for both ADHD and ASD but screen-positive for CD

	**CD screen-negative cases**	**CD screen-positive cases**	**Cases screen-negative for ADHD, ASD and CD**	**Cases screen-negative for ADHD and ASD but screen-positive for CD**
No Motor problems	15,143	530	14,101	271
	92.8%	79.7%	94.7%	94.8%
Motor problems	1,177	135	787	15
	7.2%	20.3%	5.3%	5.2%
Total	16,320	665	14,888	286
	100%	100%	100%	100%
Cross-table significance	*P* < 0.001		*P* = 1	
No Perception problems	16,135	585	14,839	283
	98.9%	88.0%	99.7%	99.0%
Perception problems	185	80	49	3
	1.1%	12.0%	0.3%	1.0%
Total	16,320	665	14,888	286
	100%	100%	100%	100%
Cross-table significance	*P* < 0.001		*P* = 0.075	

Column percentages are shown. Cross-table significances have been calculated according to Fisher’s exact test.

Children born prematurely have been shown to have a higher frequency of motor delays and motor difficulties than children born full term [40]. Since many children were born prematurely (29%) we compared the correlations between motor/perceptual problems and caseness for ADHD, ASD and CD for children born prematurely and children born full term. The correlation coefficients are given in Table 4. The only significant differences found between the correlation coefficients were those concerning the correlations between Perceptual disorder caseness and ASD caseness (*P* < 0.001), DCD caseness and CD caseness (*P* = 0.003) and Perceptual disorder caseness and CD caseness (*P* < 0.001). For perceptual disorder caseness we found that the correlation was stronger for full term children than for those born prematurely. Thus omitting prematurely born children from the analysis would strengthen the association between perceptual problems and ASD caseness. This shows that the strong correlations between caseness for ADHD and ASD and having motor and/or perceptual problems are not explicable only by the fact that many of the children were born prematurely.

**Table 4:** Spearman correlations between caseness for Developmental Coordination Disorder (DCD) and perception disorder on one hand and caseness for the different diagnoses (Attention-Deficit/Hyperactivity Disorder (ADHD), Autism Spectrum Disorder (ASD) and Conduct Disorder (CD)) on the other for children born preterm and full term born children

**Correlations between caseness for:**	**Children born preterm**	**Full term born children**	***P***
DCD/ADHD	rho = 0.25, n = 4,906	rho = 0.22, n = 12,016	0.12
DCD/ASD	rho = 0.30, n = 4,912	rho = 0.30, n = 12,024	0.92
DCD/CD	rho = 0.059, n = 4,912	rho = 0.11, n = 12,026	0.003
Perception disorder/ADHD	rho = 0.25, n = 4,906	rho = 0.26, n = 12,016	0.41
Perception disorder/ASD	rho = 0.42, n = 4,912	rho = 0.48, n = 12,024	< 0.001
Perception disorder/CD	rho = 0.078, n = 4,912	rho = 0.21, n = 12,026	< 0.001

*P* = significance of the difference between the correlations for children born preterm and full term born children.

### Neurodevelopmental and behavioural problems associated with motor or perception dysfunction

Dimensional analyses with continuous A-TAC scores were used in the binary logistic regression analyses in GEE models and the dependence of each twin in a pair was taken into consideration. Male versus female gender was associated with an odds ratio (OR) of 1.86 (95% CI = 1.64 to 2.10) and 2.34 (95% CI = 1.77 to 3.09), in the prediction of Motor control and Perception problems respectively. Age was not associated with the risk of Motor control or Perception problems.

In univariable models, the A-TAC scores of ADHD, ASD, and CD, analysed as continuous scale variables, were significantly (*P* < 0.05) positively associated with both Motor control and Perception problems in both genders (Table 5). In the multivariable model, the CD-score no longer showed significant positive associations with Motor control or Perception problems in either gender.

**Table 5:** Results according to the General Estimated Equation (GEE) model concerning the associations between Motor problems and Perception problems and scores of Attention-Deficit/Hyperactivity Disorder (ADHD), Autism Spectrum Disorder (ASD) and Conduct Disorder (CD)

**BOYS**					**Motor problems**				**Perception problems**			
**Variable**	**Crude measures**				**Univariable model**		**Multivariable model**^**a**^		**Univariable model**		**Multivariable model**	
	N	Min to Max	Mean	SD.	OR	95% CI	OR	95% CI	OR	95% CI	OR	95% CI
					B	95% CI	B	95% CI	B	95% CI	B	95% CI
ADHD	8,643	0 to 19	2.34	3.32	1.22^d^	1.20 to 1.24	1.12^d^	1.09 to 1.15	1.38^d^	1.34 to 1.42	1.12^d^	1.06 to 1.18
					0.20^d^	0.18 to 0.22	0.11^d^	0.09 to 0.14	0.32^d^	0.30 to 0.35	0.11^d^	0.06 to 0.16
ASD	8,643	0 to 17	0.91	1.71	1.53^d^	1.46 to 1.60	1.38^d^	1.30 to 1.46	1.92^d^	1.81 to 2.04	1.74^d^	1.60 to 1.89
					0.42^d^	0.38 to 0.47	0.32^d^	0.26 to 0.38	0.65^d^	0.59 to 0.71	0.55^d^	0.47 to 0.64
CD	8,643	0 to 5	0.11	0.38	1.86^d^	1.62 to 2.14	0.66^d^	0.54 to 0.80	3.12^d^	2.50 to 3.90	0.84	0.63 to 1.14
					0.62^d^	0.48 to 0.76	-0.42^d^	-0.61 to -0.23	1.14^d^	0.92 to 1.36	-0.17	-0.47 to 0.13
**GIRLS**					**Motor problems**				**Perception problems**			
ADHD	8,310	0 to 19	1.45	2.51	1.26^d^	1.23 to 1.29	1.15^d^	1.10 to 1.19	1.43^d^	1.37 to 1.49	1.22^d^	1.13 to 1.32
					0.23^d^	0.21 to 0.26	0.14^d^	0.10 to 0.18	0.36^d^	0.32 to 0.40	0.20^d^	0.12 to 0.28
ASD	8,310	0 to 14.5	0.55	1.14	1.70^d^	1.60 to 1.82	1.48^d^	1.36 to 1.62	2.00^d^	1.82 to 2.20	1.62^d^	1.42 to 1.86
					0.53^d^	0.47 to 0.60	0.40^d^	0.31 to 0.48	0.69^d^	0.60 to 0.79	0.49^d^	0.35 to 0.62
CD	8,310	0 to 4.5	0.06	0.28	2.22^d^	1.80 to 2.72	0.60^c^	0.45 to 0.81	3.70^d^	2.79 to 4.90	0.69	0.42 to 1.13
					0.80^d^	0.59 to 1.00	-0.50^c^	-0.80 to -0.21	1.31^d^	1.03 to 1.59	-0.38	-0.87 to 0.12

^a^Multivariable models are controlled for age which in univariable models was not significantly associated with the dependent variables.

B coefficients and odds ratios (OR) for the univariable model and adjusted B coefficients and odds ratios for the multivariable model with their 95% confidence intervals (CI) are shown.

^b^0.01 ≤ *P* < 0.05; ^c^0.001 ≤ *P* < 0.01; ^d^*P* < 0.001.

ADHD = Attention Deficit/Hyperactivity Disorder; ASD = Autism Spectrum Disorder; CD = Conduct Disorder.

The subdomains of ADHD (Concentration/Attention, Impulsiveness/Activity) and the subdomains of ASD (Language, Social Interaction and Flexibility) were analysed as continuous scale variables in a multivariable model together with the continuous CD-score (Table 6). In this analysis, the score of Impulsiveness/Activity and the score of CD had no significant positive associations with Motor control or Perception problems in either gender, and the score of Social Interaction had a significant (*P* < 0.05) association with Motor control problems only in boys. The strongest positive significant association in both genders was that between Flexibility and Motor control or Perception problems (Table 6).

**Table 6:** Results according to the General Estimated Equation (GEE) model concerning the associations between motor problems and perception problems and scores of Conduct Disorder (CD) and of the subdomains of Attention-Deficit/Hyperactivity Disorder (ADHD) and Autism Spectrum Disorder (ASD)

**BOYS**					**Motor problems**				**Perception problems**			
**Variable**	**Crude measures**				**Univariable model**		**Multivariable model**		**Univariable model**		**Multivariable model**	
	N	Min to Max	Mean		OR	95% CI	OR	95% CI	OR	95% CI	OR	95% CI
				SD	B	95% CI	B	95% CI	B	95% CI	B	95% CI
Concentration and Attention	8,631	0 to 9	1.24	1.89	1.47^d^	1.43 to 1.52	1.33^d^	1.27 to 1.39	1.82^d^	1.73 to 1.92	1.30^d^	1.18 to 1.43
					0.39^d^	0.36 to 0.42	0.28^d^	0.24 to 0.33	0.60^d^	0.55 to 0.65	0.26^d^	0.16 to 0.36
Impulsiveness and Activity	8,631	0 to 10	1.09	1.79	1.30^d^	1.27 to 1.35	0.93^b^	0.88 to 0.98	1.63^d^	1.55 to 1.71	0.97	0.88 to 1.08
					0.27^d^	0.78 to 0.95	-0.07^b^	-0.12 to 0.02	0.49^d^	0.44 to 0.53	-0.03	-0.13 to 0.08
Flexibility	8,631	0 to 5	0.31	0.67	2.38^d^	2.19 to 2.60	1.34^d^	1.18 to 1.53	4.85^d^	4.23 to 5.56	2.33^d^	1.92 to 2.83
					0.87^d^	0.78 to 0.95	0.30^d^	0.17 to 0.43	1.58^d^	1.44 to 1.72	0.84^d^	0.65 to 1.04
Social Interaction	8,631	0 to 6	0.31	0.67	2.35^d^	2.14 to 2.58	1.18^b^	1.01 to 1.37	3.99^d^	3.50 to 4.56	1.42^c^	1.15 to 1.75
					0.85^d^	0.76 to 0.95	0.16^b^	0.01 to 0.31	1.39^d^	1.25 to 1.52	0.35^c^	0.14 to 0.56
Language	8,631	0 to 6	0.29	0.66	2.68^d^	2.44 to 2.95	1.63^d^	1.44 to 1.84	3.98^d^	3.49 to 4.53	1.49^d^	1.23 to 1.81
					0.99^d^	0.89 to 1.08	0.49^d^	0.36 to 0.61	1.38^d^	1.25 to 1.51	0.40^d^	0.21 to 0.60
CD	8,631	0 to 5	0.11	0.38	1.86^d^	1.62 to 2.15	0.77^b^	0.64 to 0.94	3.16^d^	2.53 to 3.94	0.94	0.70 to 1.26
					0.62^d^	0.48 to 0.76	-0.26^b^	-0.45 to -0.06	1.15^d^	0.93 to 1.37	-0.06	-0.36 to 0.23
**GIRLS**					**Motor problems**				**Perception problems**			
Concentration and Attention	8,296	0 to 9	0.73	1.43	1.54^d^	1.47 to 1.60	1.38^d^	1.29 to 1.47	1.97^d^	1.82 to 2.12	1.57^d^	1.38 to 1.77
					0.43^d^	0.38 to 0.47	0.32^d^	0.25 to 0.38	0.68	0.60 to 0.75	0.45	0.33 to 0.57
Impulsiveness and Activity	8,296	0 to 10	0.71	1.40	1.36^d^	1.30 to 1.42	0.95	0.88 to 1.03	1.68^d^	1.56 to 1.80	0.96	0.82 to 1.12
					0.31^d^	0.26 to 0.35	-0.05	-0.13 to 0.03	0.52	0.45 to 0.59	-0.04	-0.20 to 0.11
Flexibility	8,296	0 to 5	0.16	0.45	2.99^d^	2.62 to 3.42	1.79^d^	1.50 to 2.15	4.70^d^	3.87 to 5.70	1.78^d^	1.33 to 2.37
					1.10^d^	0.96 to 1.23	0.58^d^	0.40 to 0.77	1.55	1.35 to 1.74	0.58	0.29 to 0.86
Social Interaction	8,296	0 to 5.5	0.20	0.50	2.59^d^	2.28 to 2.93	1.17	0.95 to 1.44	4.30^d^	3.62 to 5.10	1.54^c^	1.11 to 2.14
					0.95	0.83 to 1.08	0.16	-0.06 to 0.37	1.46	1.29 to 1.63	0.43	0.11 to 0.76
Language	8296	0 to 5	0.18	0.46	2.88^d^	2.52 to 3.30	1.54^d^	1.29 to 1.83	4.22^d^	3.42 to 5.21	1.49^b^	1.06 to 2.09
					1.06^d^	0.92 to 1.19	0.43^d^	0.26 to 0.60	1.44	1.23 to 1.65	0.40	0.06 to 0.74
CD	8296	0 to 4.5	0.06	0.28	2.19^d^	1.78 to 2.69	0.65^c^	0.48 to 0.87	3.68^d^	2.77 to 4.89	0.74	0.44 to 1.22
					0.78^d^	0.58 to 0.99	-0.43^c^	-0.73 to -0.14	1.30	1.02 to 1.59	-0.31	-0.81 to 0.20

^a^Multivariable models are controlled for age which in univariable models was not significantly associated with the dependent variables.

B coefficients and ORs for the univariable model and adjusted B coefficients and ORs for the multivariable model with their 95% CI are shown.

^b^0.01 ≤ *P* < 0.05; ^c^0.001 ≤ *P* < 0.01; ^d^*P* < 0.001.

CD = Conduct Disorder.

## Discussion

This is probably the largest study ever performed on the relationship between symptoms relating to commonly diagnosed problems in child and adolescent psychiatry - ADHD, ASD, and, CD - and motor control and perception problems - commonly encountered but often not separately diagnosed, either as DCD or under any other label. ADHD and ASD, as ascertained on the basis of parent telephone interviews, showed strong and independent associations with such DCD-related problems, but CD without ‘comorbid’ ADHD or ASD did not. Associations were established both when phenomena were studied on a categorical basis and when they were considered from the point of view of continuous distributions. Parents were interviewed using the A-TAC, an instrument which is used for easy and quick detection of childhood psychiatric problems [41]. Data from 16,994 parent interviews were used, which gives the study good statistical power.

The hypothesis that there is an association between CD and motor and/or perceptual problems was not supported by our study. The results seem to indicate that there are different forms of CD. One type of CD is associated with attention deficit and/or autistic symptoms and these children have a high frequency of motor and/or perceptual problems indicating a more ‘organic’ kind of disturbance. Another type of CD is associated with hyperactive and impulsive behaviour and children in this group have a low frequency of motor and/or perceptual problems and neither attention deficit nor autistic symptoms. Children with CD without either ADHD-symptoms or autistic symptoms also have a low frequency of motor and/or perceptual problems. Thus motor and/or perceptual problems among children with CD should lead a clinician to suspect comorbid attention deficit and/or autistic symptoms which might be of importance in efforts to find the right treatment and support.

ADHD is often argued to be a heterogeneous condition with different subtypes; for example, the DAMP subtype of ADHD with motor and perception problems [3]. Lambek *et al*. [42] have described one group of children with lower IQ and an executive function deficit, and another group with normal IQ and no signs of an executive function deficit but with delay aversion (difficulty waiting for a reward), which can be regarded as a kind of lack of impulse control. Our study seems to indicate the existence of one group of children with high scores of ADHD but low scores of ASD and without Motor control or Perception problems, some of them with high scores of Impulsiveness/Activity and CD; and another group of children with high scores of ADHD and ASD but with low scores of Impulsiveness/Activity and CD and with Motor control and Perception problems. ASD has also been suggested to be heterogeneous in the sense that the three symptom groups in the ‘triad’ of ASD, that is social interaction difficulties, communication difficulties, and behaviour symptoms, have been shown to have limited genetic and environmental overlap, the so called ‘fractionable autism triad hypothesis’ [43]. Robinson *et al*. [43] have performed a twin study where they could show that the majority of genetic influences on autism-like behaviours are distinct to each phenotypical component, thus giving support to the ‘fractionable autism triad hypothesis’. Different components of the ASD phenotype might be associated with different etiologic factors. Since many studies indicate that both ADHD and ASD can be regarded as heterogeneous conditions, we decided to analyse the different symptoms of ADHD and ASD separately. Of the two subdomains of ADHD (Concentration/Attention and Impulsiveness/Activity) only Concentration/Attention had significant associations with Motor control and Perception problems, whereas all three subdomains of ASD (Flexibility, Social Interaction and Language) were associated with perceptual problems in both boys and girls and with motor problems in boys and girls, with the exception of Social Interaction in girls.

Cerebellar function is of importance for motor function and seems to be of importance in ASD as well as in ADHD [23,44]. The association between CD and cerebellar function among children without comorbid ADHD or ASD has not been studied. A Swedish study of regional cerebral blood flow using SPECT (Single Photon Emission Computed Tomography) in children with ADHD showed two different non-correlated patterns; one associated with motor impairment and cognitive function (low blood flow in the cerebellum and temporal lobes relative to the basal ganglia and thalamus) and one associated with deviant behaviour (low blood flow in the frontal and parietal areas) [45]. One might hypothesise that the children in our study with a high score of ADHD, a low score of ASD and who had no Motor control problems may have cerebellar function more comparable with children without a neurodevelopmental disorder, whereas the other group with high scores of ADHD and ASD and with Motor control and Perception problems may have relative cerebellar dysfunction. The first group may share some characteristics with the group without an executive function deficit described by Lambek *et al*. [42], while the other group may share characteristics with the group with lower IQ and an executive function deficit.

### Limitations

The assessment of Motor control and Perception problems and of screen positivity for ADHD, ASD and, CD was based on information from parent interviews. In this study, we could not examine the children or obtain information from the teacher as in a thorough clinical assessment. Depending on only one subjective source of information is a weakness. The parents’ description of the child’s motor and perception functions and the result of a neurological examination of the child have been shown to have a low to moderate degree of correlation [46]. On the other hand, the parental interview used in the present study, the A-TAC, has shown excellent diagnostic utility for identifying ADHD and fair diagnostic utility for diagnosing DCD [39]. The study is a part of a national twin study. Although twins are not completely representative of the normal population in terms of mental health problems and psychomotor development, and some bias may be introduced by this, most studies that have examined this issue have found no or very small differences between twins and singletons [47].

## Conclusions

Meeting criteria for CD caseness was positively associated with Motor control and/or Perception problems only in individuals who simultaneously met criteria for ADHD caseness and/or ASD caseness. High scores of the continuous variables Impulsiveness/Activity or CD in the absence of high scores of any of the variables Concentration/Attention, Flexibility, Social Interaction, or Language were not associated with Motor control and/or Perception problems. Thus, CD symptoms or problems with Impulsiveness/Activity seem to be associated with Motor control or Perception problems only in the presence of ASD symptoms and/or symptoms of inattention. This seems to indicate that children with CD without ASD or inattention do not show a deviant development of motor and perceptual functions. Children with CD should be examined concerning motor function and perception, and a suspicion of attention deficit disorder and/or ASD should be raised if the child is found to have motor and/or perceptual problems.

## Consent

Written informed consent was obtained from the patient’s guardian/parent/next of kin for the publication of this report.

## Abbreviations

ADHD: Attention-Deficit/Hyperactivity Disorder; ASD: Autism Spectrum Disorder; A-TAC: The Autism-Tics ADHD and other Comorbidities inventory; CATSS: Child and Adolescent Twin Study in Sweden; CD: Conduct Disorder; CI: confidence interval; DAMP: Deficits in Attention Motor control and Perception; DCD: Developmental Coordination Disorder; DSM-IV/5: *Diagnostic and Statistical Manual of Mental Disorders 4th edition/5th edition*; GEE: General Estimated Equation models; MBD: Minimal Brain Dysfunction/Damage; OR: odds ratio; SPECT: Single Photon Emission Computed Tomography.

## Competing interests

All authors declare that we have no competing interests.

## Authors’ contributions

PG was responsible for the design of the study, performed statistical analyses and drafted the manuscript. NK participated in the design of the study, performed statistical analyses and helped to draft the manuscript. HA, PL, CG and MR came with advice when interpreting and describing the results and helped to draft the manuscript. All authors read and approved the final manuscript.
